# Housing, Living Arrangements and Mental Health of Young Adults in Independent Living

**DOI:** 10.3390/ijerph18105250

**Published:** 2021-05-14

**Authors:** Bo-Kyong Seo, Gum-Ryeong Park

**Affiliations:** 1 Department of Applied Social Sciences, The Hong Kong Polytechnic University, Hung Hom, Hong Kong; 2 Department of Health, Aging & Society, McMaster University, Hamilton, ON L8S 4L8, Canada; parkg9@mcmaster.ca; 3 Department of Health Care Policy Research, Korea Institute for Health and Social Affairs, Sejong 30147, Korea

**Keywords:** housing cost burden, perceived housing quality, living arrangements, mental health, young adults

## Abstract

Young adults are prone to psychological stress and anxiety induced by major transitions to adulthood. While employment has predominated in previous research on the social determinants of young people’s mental health, this study examines the association between young people’s housing problems and mental health in the context of an unaffordable housing market. Using the Survey on the Living Conditions and Welfare Needs of Youths (*n* = 1308) in Korea, the study found that perceived poor housing quality and material hardship are negatively associated with the mental health of young adults living independently. Specifically, while poor housing quality and material hardship induced by housing cost burden were negatively associated with single-person households’ mental health, only poor housing quality was associated with non-single-person households’ mental health. This study is one of the few studies examining the linkage between housing problems and mental health of young adults and informs the interventions aimed at promoting the psychological well-being of young adults in the transition from parents’ homes to independent living.

## 1. Introduction

Young people are generally defined as those aged late teens to 24 [1]. They usually experience major transitions to adulthood in terms of education, employment and social identity that are likely to engender uncertainty about their future [2,3,4]. Yet, independent living, marriage and parenthood have increasingly been delayed among young adults in the face of the neoliberal labor market, and hence, the age threshold for the sociological notion of young people has been extended to the early 30s [5].

The literature has reported that life transitions to adulthood influence mental health among young people [5,6,7]. Developmental studies suggest that young adults’ mental health is largely affected by the social context, rather than biological factors [3]. Gaining stability in the social context through employment, social support and marriage has been shown to be associated with the reduced depressive disorders among young adults [8]. In particular, the adverse effect of insecure employment has been well established in previous research on young people’s psychological well-being [9,10,11].

However, housing factors have received surprisingly little attention as a social determinant of mental health among young adults, particularly those in transition from a family home to independent living. While private rental is the most common tenure type in the early stage of independent living, it has increasingly been challenging for young people to access adequate and affordable housing due to the constantly rising housing prices [6,12,13,14,15,16]. A growing body of research has reported on delayed homeownership [17], increased house sharing [18], frustrated housing prospects [19] and material hardship [2] among young people today. Previous research focusing on children, older persons and low-income families found that an inadequate housing environment, housing cost burden, being a renter and insecure tenancy are associated with poor mental health [20,21,22,23,24,25,26,27]. However, the effect of these housing problems on young people’s mental health has been largely understudied [4,28]. Only a few researchers have explored the link between housing-related factors, such as eviction [29] and house sharing [30], and poor mental health of young adults. To fill the gap in the literature, this study examines, from an ecological perspective, how housing problems are associated with the mental health of young people, using the Korean dataset on young adults.

In the Republic of Korea (Korea hereafter), extended young adulthood and poor mental health have increasingly become social problems in recent years. Many young Koreans aged between 19 and 34 tend to delay entering the job market after graduation from university and subsequently postpone marriage and child bearing [31,32]. They have also shown a higher level of perceived stress compared to other age groups in Korea since the global economic crisis in 2008 [33]. According to the latest National Housing Survey [34], 59.2% of Koreans aged 20 to 34 are single-person households (nation 29.3%), and 17.2% are owner–occupiers (nation 58%). About 37% of single-person households among the young population in Seoul live in substandard housing conditions, basements, rooftops or non-residential buildings due to deteriorating housing affordability [35]. Therefore, young people’s housing difficulties in Korea are likely to affect their mental health, and yet this relation has not been elucidated.

In this paper, we aim to answer the following four specific research questions. First, to what extent are the quality of housing environment and housing cost burden associated with the mental health of young people? Given the generally significant relation between perceived housing quality and residents’ mental health [36,37], we pay attention to young adults’ perceived housing quality, rather than objective elements, to capture the variation of the quality of housing environment in Korea. Second, does the exposure to multiple housing problems have stronger associations with depressive symptoms? Applying a cumulative risk model which has been widely adopted in research investigating risk factors for mental health [38,39], we assume that the more housing problems young people are exposed to, the greater the likelihood of having mental health problems [40]. Third, is the association between housing cost burden and mental health mediated by material hardship that young people face? This question draws on previous research suggesting that housing problems and material hardship are often observed concomitantly among young people [41,42]. Finally, does the association between housing problems and the mental health of single-person households differ from that of non-single-person households? The literature suggests that living with someone else is conducive to seniors’ mental health [43,44], but little is known about how it affects young adults.

## 2. Methods

### 2.1. Dataset

This study used the dataset from the Survey on the Living Conditions and Welfare Needs of Youths. The survey was conducted by the Korea Institute for Health and Social Affairs in 2019, in preparation for the government’s legislation of the Framework Act on Youth in August 2020. Targeting young adults aged 19 to 34, it collected information about the socioeconomic status, demographic characteristics and welfare needs of 3018 households. In view of the generally high proportion of single-person households in Korea (29.3%), this survey differentiated the types of living arrangement of young adults into single-person households, young people who do not live with their parents and young people who live with their parents. As the housing status of young people who co-reside with their parents is determined by their parents’ socioeconomic status and parental housing characteristics, we limited the participants to those who lived independently from their parents. The final sample included 1308 households that comprised 761 single-person households and 547 non-single-person households (i.e., those who lived with their own partners, friends or flat mates). Ethical approval was not required for this study since the data were publicly available (https://data.kihasa.re.kr, accessed on 15 March 2021).

### 2.2. Measures

#### 2.2.1. Depressive Symptoms

To measure young adults’ mental health, we used the 11 items from the Center for Epidemiological Studies Depression (CES-D) scale included in the survey. CES-D is a reliable and validated scale to measure depressive symptoms, which has been widely used in previous studies, e.g., [45,46]. The scale asked the study participants how often they experienced feelings of those 11 items during the past week using the possible responses ranging from 0 (very rare or less than a day) to 3 (almost all of the time or more than 5 days). The 11 items include: during the last week, (a) I did not feel like eating and my appetite was poor; (b) I felt that I was doing generally well; (c) I felt depressed; (d) I had trouble keeping my mind on what I was doing; (e) I could not sleep well; (f) I felt lonely; (g) I went on without much complaints; (h) I felt that people were treating me coldly; (i) I felt sad; (j) I felt that people disliked me; and (k) I was unable to have the courage to carry out something’. By adjusting all the responses in a way that the aggregated scores ranged from 0 to 20, after reversely coding the two items (b) and (g), we defined participants with a score of over 16 as having depressive symptoms.

#### 2.2.2. Housing Problems

For housing cost burden, we used 30% of household income as the housing affordability threshold that is widely used in the housing literature, e.g., [41]. Household income includes earnings and cash transfers from the government, and housing costs include monthly rent, mortgage principal and interest repayment and maintenance costs. We defined the participants whose housing costs exceeded 30% of their household income as having housing cost burden (=1), with all others falling under 0.

We used young people’s self-assessment of the functional characteristics of their accommodations to measure perceived housing quality. The participants responded from ‘very poor (=1)’ to ‘very good (=4)’ to eight categories of the housing environment: lighting, soundproof, dampness, ventilation, protection from break-in, protection from natural disaster, protection from fire, and sanitation. If the participants answered ‘very poor’ or ‘poor’, their housing units were considered in inadequate quality regarding the particular category (=1), or otherwise (=0). Then, we added up the scores; a higher score indicates poorer perceived quality of housing environment. In examining the association between the breadth of housing problems and depressive symptoms, we considered the participants whose score was 1 or above as having ‘inadequate housing quality’ (=1) and otherwise (=0).

#### 2.2.3. Material Hardship

We measured three types of material hardship to which the study participants were required to answer: (a) Have you ever been forced to reduce food expenditure due to housing cost burden in the past year? (b) Have you ever not been able to use air-conditioning system due to economic difficulties in the past year? (c) Have you ever not been able to use heating system due to economic difficulties in the past year? For those who answered ‘very often’ or ‘often’ to either of these questions, we constructed a binary indicator of material hardship (=1), and no material hardship (=0) if they answered ‘very rare’ or ‘none’.

#### 2.2.4. Control Variables

We checked the moderation effects of the respondents’ socioeconomic characteristics, such as age (19–24, 25–29, 30 and over), gender (male, female), educational attainment (university/college or less, university/college graduate or higher), quintile household income, employment status (full time, part time, economically inactive and unemployed), tenure (owner occupiers, renters, others), and city of residence (urban, rural) on the association between housing problems and mental health and confirmed that there was no moderation effect. Therefore, these variables were included as covariates for the analysis.

### 2.3. Statistical Analysis

First, we examined the general characteristics of the study participants and the prevalence of depressive symptoms by housing problems, i.e., inadequate housing quality and housing cost burden. Then, we conducted a series of logistic regression analyses for the study participants who were further stratified by living arrangement type (single-person household vs. non-single-person household). To assess whether a mediating variable explains a significant amount of the relationship between housing cost burden and depressive symptoms, we conducted mediation tests, following Baron and Kenny [47]. The odds ratios (ORs) were adjusted for covariates, and ORs and 95% confidential intervals (95% CI) were presented in the results. However, if a binary outcome is common (usually 10% as a cutoff), ORs with mediator and ORs without mediator might not be completely comparable [48]. This is because adding covariates in logistic regression is likely to increase the magnitude of the coefficient. Therefore, drawing on VanderWeele [48], we used a log-linear model, instead of a logistic model, for mediation analysis to avoid potential errors. STATA/SE version 15.0 was used for all analyses.

## 3. Results

### 3.1. Prevalence of Housing Problems and Depressive Symptoms

Table 1 presents the distribution of housing problems and depressive symptoms of the study sample. It showed that 85.2% experienced housing problems, and it was more prevalent among single-person households (87.9%) than non-single-person households (83.2%). While 50.9% had only either inadequate housing quality or housing cost burden, 34.3% suffered both housing problems. While the prevalence of housing cost burden was higher among non-single-person households (81.7%) compared to single-person households (69.4%), single-person households had a higher prevalence of inadequate housing environment (47.2%) compared to the counterpart (41.9%). The average number of items of the housing quality (out of eight items) that the respondents perceived as inadequate was 1.14 (SD: 1.72), which was higher among single households (1.22, SD: 1.79) compared to non-single households (1.01, SD: 1.61). As for the different types of inadequate housing environment, poor soundproofing was most prevalent (37.2%), followed by dampness (15.5%) and poor protection from break-in (11.5%). While 21.5% experienced material hardship, the prevalence was higher among single-person households (24.1%) compared to non-single-person households (17.9%). Overall, 12.4% indicated depressive symptoms, and the prevalence was higher among single-person households (14.6%) than non-single-person households (9.3%).

### 3.2. The Association between Housing Problems and Depressive Symptoms

Table 2 indicates the association between housing cost burden, inadequate housing quality and depressive symptoms among young adults. It shows that the number of items of the housing quality that the respondents perceived as inadequate was significantly associated with depressive symptoms in the entire sample (OR: 1.318, 95% CI: 1.215–1.429), whereas housing cost burden did not predict depressive symptoms (OR: 1.470, 95% CI: 0.947–2.283) after adjusting for covariates. When stratified by household type, both housing cost burden (OR: 1.916, 95% CI: 1.134–3.236) and the number of items of the housing quality perceived as inadequate (OR: 1.313, 95% CI: 1.188–1.451) were significantly associated with depressive symptoms among single-person households, and only the number of items of the housing quality perceived as inadequate was significantly associated with depressive symptoms among non-single-person households (OR: 1.338, 95% CI: 1.154–1.551).

### 3.3. The Association between the Breadth of Housing Problems and Depressive Symptoms

The association of the breadth of housing problems with depressive symptoms is presented in Table 3. Compared to households without any of the housing problems, households who experienced a single housing problem were 2.239 times more likely to have depressive symptoms (95% CI: 1.121–4.470), and those who had double housing problems were 4.339 times more likely to have depressive symptoms (95% CI: 2.152–8.744). Single-person households showed a pattern similar to that of the entire study sample (with a single housing problem OR: 3.445, 95% CI: 1.435–8.269; with double housing problems OR: 5.573, 95% CI: 2.282–13.608), after adjusting for covariates. However, we did not find a significant association between the breadth of housing problems and depressive symptoms among non-single-person households.

### 3.4. The Mediating Effect of Material Hardship on the Association between Housing Cost Burden and Depressive Symptoms

The mediation effect of material hardship was examined to see if it links housing cost burden and depressive symptoms. Table 4 shows that when housing cost burden was regressed on material hardship, the association was significant among all study participants (OR: 5.659, 95% CI: 2.976–10.761) and single-person households (OR: 2.195, 95% CI: 1.442–3.344), but insignificant among non-single-person households (OR: 1.977, 95% CI: 0.938–4.164). As explained in the ‘Methods’ section above, we conducted log-linear regression for mediation analysis and found that the significant effect of housing cost burden on depressive symptoms among single-person households became insignificant (coefficient: 0.035, 95% CI: −0.004–0.074) when material hardship was included in Model 2 (Table 5). According to Baron and Kenny [47], if an independent variable is no longer significant when a mediating factor is controlled for, the model supports full mediation. This result is consistent with the result of the logistic model (for the result of logistic regression, see Table S1 in the supplementary material). Therefore, we conclude that material hardship is a potential mediator, particularly for single-person households. Meanwhile, the association between material hardship and depressive symptoms was significant in the entire sample (coefficient: 0.010, 95% CI: 0.074–0.134 in the log-linear model; OR: 1.232, 95% CI: 0.794–1.911 in the logistic model), while housing cost burden was not significantly associated with depressive symptoms of the entire sample. This result indicates that material hardship is another predictor of depressive symptoms for all participants, rather than a mediator linking housing cost burden and mental health.

## 4. Discussion

Our analysis showed that a number of young Koreans in independent living experienced housing cost burden, inadequate housing quality and material hardship, similar to what has been found in other studies, e.g., [41,42]. This result strengthens the evidence for the housing crisis that young people in Korea have encountered recently. All in all, our study demonstrates that perceived poor housing quality and material hardship are negatively associated with the mental health of young adults living independently. This finding proves that the association between housing quality and mental health among older people and low-income families [49,50] is shown in a similar manner among young adults. In addition, it was demonstrated that the more housing problems young people are exposed to, the greater risks of mental health problems they are prone to, which supports the foundational assumption of the cumulative risk model [39,51]. Moreover, since all the analyses in our study controlled for young people’s working status, the significant association between housing problems and depressive symptoms indicates that housing matters for young people’s mental health in its own right. This result strengthens the importance of the ecological context in understanding risk factors for young people’s mental health, which have been examined predominantly from the employment-centered point of view [9,10,11].

What is more interesting is that single-person households and non-single-person households showed notably different associations between housing problems and mental health. We found that the relations between housing problems and mental health among single-person households are more in accordance with most research that proved the negative effects of poor housing quality and unaffordable housing on mental health e.g., [21,25]. In addition, we also noted that housing cost burden forces young adults in single-person households to scrimp on their budget for other basic needs, such as food and energy consumption, which in turn has a negative effect on their depressive symptoms. In stark contrast, however, non-single-person households’ mental health was associated only with inadequate housing quality, not by housing cost burden or material hardship, despite the larger proportion of housing cost burden among non-single-person households (81.7%) than single-person households (69.3%) (Table 1). In short, while perceived housing quality is a significant determinant of young people’s mental health regardless of their living arrangement, housing cost burden is detrimental only to single-person households.

Our further analysis showed that the insignificant effect of housing cost burden on non-single-person households’ mental health was not attributed to their homeownership status or relatively higher income level since the observed association was not significant in univariate analysis before multivariate analysis (see Table S2 in the supplementary material). Yet, there are some possible explanations. From a psychological development perspective, our study supports that co-living or forming one’s own family is beneficial to the psychological well-being of young people in transition to adulthood [3,52], although they might have to endure housing unaffordability. From an ecological perspective, this result is possibly because non-single-person households could enjoy more gross/net floor spaces and more favorable internal housing conditions than single-person households, as they tend to share a housing unit that has both private rooms and communal spaces. Considering that a number of single-person households, particularly in Seoul, seek inadequate and unsafe studio-type flats or subdivided units due to their affordability [53], the relatively better housing quality of non-single-person households might account for the insignificant effect of housing cost burden on non-single-person households’ mental health. That is, even though young adults need to pay more than 30% of their household income for housing, it may not affect their mental health adversely if the housing quality is satisfactory. Although housing size was not included in our analysis, the lower prevalence of perceived poor housing quality among non-single-person households than single-person households (Table 1) partly helped us understand this result. This finding seems to advance the existing literature on the relationship between living arrangements and young adults’ mental health [54,55], which tends to overlook the ecological factors.

## 5. Conclusions

This study, one of the few studies examining the linkage between housing problems and mental health of young adults in independent living, strengthens the significance of housing as a social determinant of young people’s mental health. This study broadens the discussions on the relation between housing and mental health by taking both housing environment and housing affordability dimensions into account in examining mental health effects and differentiating the household types, i.e., single household and non-single household, that might have different mental health implications. The negative association of perceived poor housing quality and housing cost burden with the mental health of young adults, with employment controlled for, affirms the findings of previous studies on other socioeconomically vulnerable groups. The distinction between single-person households and non-single-person households implies that living arrangement and family formation influences the effect of housing affordability on young people’s mental health. Nevertheless, our study has its limitations. As the dataset we used was cross-sectional, the causal inference of the results was unclear. For example, it is possible that households with depressive symptoms are likely to experience housing problems. In addition, the relatively small sample size of each subset group, i.e., single-person households vs. non-single-person households, refrained from further categorization into more detailed household and housing types within each subset group, which might have helped provide a richer account of the result.

Our study informs the interventions aimed at promoting the psychological well-being of young adults in transition from parents’ home to independent living. It implies that improving the environmental quality standards and retrofitting of housing types that are popular among young adults in the early stage of education or career would be conducive to young adults’ mental health. Public assistance that can help reduce young people’s financial hardship induced by housing cost burden would also contribute to young adults’ mental health. In this regard, the Korean government’s recent policy change to implement subsidized housing programs targeting university students, early career workers and newlyweds [56] and separate unmarried young people in independent living from their parents’ households for housing benefit distribution [57] seems to not only enhance young people’s housing quality but also reduce their potential mental health risks. Considering the distinction between single-person households and non-single-person households in the effect of housing cost burden on mental health, co-living/shared housing that has mushroomed as a popular accommodation for single young persons in some developed Asian countries with unaffordable housing markets, including Korea, might be worth investigating in the future studies with regard to its mental health effect.

## Figures and Tables

**Table 1:** Descriptive statistics of the study population.

	Total (*n* = 1308)		Types of Household			
			Single-Person(*n* = 761)		Non-Single-Person (*n* = 547)	
	Distribution (%)	Depressive Symptoms (%)	Distribution (%)	Depressive Symptoms (%)	Distribution (%)	Depressive Symptoms (%)
Type of housing problem						
Housing cost burden	74.5	13.3	69.3	16.5	81.7	9.6
Inadequate housing quality ^1^	45.0	17.5	47.2	19.2	41.9	14.8
Poor lighting	10.2	22.6	13.3	24.8	5.9	15.6
Poor soundproof	37.2	18.3	38.5	20.1	35.3	15.5
Dampness	15.5	19.7	14.7	23.2	16.6	15.4
Poor ventilation	10.1	28.0	12.6	29.2	6.6	25.0
Poor protection from break-in	11.5	24.0	11.7	27.0	11.2	19.7
Poor protection from natural disaster	9.4	27.6	10.4	27.8	8.0	27.3
Poor protection from fire	10.6	25.9	11.3	31.4	9.7	17.0
Poor sanitization	9.2	23.3	9.9	26.7	8.2	17.8
Number of housing problem						
Single	50.9	10.7	49.4	14.2	52.3	5.9
Double	34.3	18.1	33.3	20.2	35.7	15.4
At least one of them	85.2	13.6	87.9	16.6	83.2	9.8
Material hardship	21.5	24.6	24.1	24.6	17.9	24.5
Food insecurity	17.9	24.4	20.0	23.7	15.0	25.6
Not being able to use heating	5.8	40.8	6.2	44.7	5.3	34.5
Not being able to use air-conditioning	6.8	38.2	7.8	39.0	5.5	36.7
Total	100.0	12.4	100.0	14.6	100.0	9.3

^1^ Households with any type of inadequate housing quality among the eight items.

**Table 2:** Association between housing problems and depressive symptoms.

	Total (*n* = 1308)		Single-Person (*n* = 761)		Non-Single-Person (*n* = 547)	
	OR	95% CI	OR	95% CI	OR	95% CI
Housing cost burden (ref: no)	1	(reference)	1	(reference)	1	(reference)
Yes	1.470	(0.947–2.283)	1.916 *	(1.134–3.236)	1.019	(0.430–2.413)
Number of items of inadequate housing quality	1.318 ***	(1.215–1.429)	1.313 ***	(1.188–1.451)	1.338 ***	(1.154–1.551)

Adjusted for sex, age, educational attainment, working status, income level, tenure, and region. * *p* < 0.05, *** *p* < 0.001.

**Table 3:** Association between the breadth of housing problems and depressive symptoms.

	Total (*n* = 1308)		Single-Person (*n* = 761)		Non-Single-Person (*n* = 547)	
	OR	95% CI	OR	95% CI	OR	95% CI
None	1	(reference)	1	(reference)	1	(reference)
Single ^1^	2.239 *	(1.121–4.470)	3.445 **	(1.435–8.269)	0.923	(0.282–3.011)
Double ^2^	4.339 ***	(2.152–8.744)	5.573 ***	(2.282–13.608)	2.808	(0.858–9.200)

Adjusted for sex, age, educational attainment, working status, income level, tenure, and region. * *p* < 0.05, ** *p*<0.01, *** *p* < 0.001. ^1^ Either housing cost burden or any types of inadequate housing quality. ^2^ Both housing cost burden and any types of inadequate housing quality.

**Table 4:** Association between housing cost burden and material hardship.

	Total (*n* = 1308)		Single-Person (*n* = 761)		Non-Single-Person (*n* = 547)	
	OR	95% CI	OR	95% CI	OR	95% CI
Housing cost burden	5.659 ***	(2.976–10.761)	2.195 ***	(1.442–3.344)	1.977	(0.938–4.164)

Adjusted for sex, age, educational attainment, working status, income level, tenure, and region. *** *p* < 0.001.

**Table 5:** Log-linear model: association between housing cost burden, material hardship and depressive symptoms.

	Total (*n* = 1308)				Single-Person (*n* = 761)				Non-Single-Person (*n* = 547)			
	Model 1		Model 2		Model 1		Model 2		Model 1		Model 2	
	Coeff.	95% CI	Coeff.	95% CI	Coeff.	95% CI	Coeff.	95% CI	Coeff.	95% CI	Coeff.	95% CI
Housing cost burden	0.024	(−0.005, 0.054)	0.013	(−0.016, 0.043)	0.047 *	(0.007, 0.086)	0.035	(−0.004, 0.074)	0.002	(−0.046, 0.050)	−0.009	(−0.055, 0.038)
Material hardship			0.010 ***	(0.074, 0.134)			0.088 ***	(0.048, 0.129)			0.131	(0.088, 0.175)

Adjusted for sex, age, educational attainment, working status, income level, tenure, and region. * *p* < 0.05, *** *p* < 0.001.

## Data Availability

The data are available at https://data.kihasa.re.kr (accessed on 15 March 2021).

## Supplementary Materials

The following are available online at https://www.mdpi.com/article/10.3390/ijerph18105250/s1, Table S1. Logistic regression: Associations between housing cost burden, material hardship and depressive symptoms; Table S2. Univariate analysis: Associations between housing cost burden, material hardship and depressive symptoms.
