# Supplementary material for: Housing, Living Arrangements and Mental Health of Young Adults in Independent Living
Source: Int J Environ Res Public Health. 2021 May 14;18(10):5250. doi: 10.3390/ijerph18105250 (PMC8156951; doi:10.3390/ijerph18105250)
Supplement: Supplementary file 1 [file ijerph-18-05250-s001.zip › ijerph-1197281-supplementary.pdf]

Table S1. Logistic regression: Associations between housing cost burden, material hardship and depressive symptoms.

	Total (n = 1308)				Single-person (n = 761)				Non-single-person (n = 547)			
	Model 1		Model 2		Model 1		Model 2		Model 1		Model 2	
	OR	95% CI	OR	95% CI	OR	95% CI	OR	95% CI	OR	95% CI	OR	95% CI
Housing cost burden (No)	1	(ref.)	1	(ref.)	1	(ref.)	1	(ref.)	1	(ref.)	1	(ref.)
Yes	1.449	(0.937-2.241)	1.232	(0.794-1.911)	1.830 *	(1.093-3.060)	1.599	(0.956-2.674)	1.030	(0.433-2.448)	0.814	(0.334-1.986)
Material hardship (No)			1	(ref.)			1	(ref.)			1	(ref.)
Yes			3.168 ***	(2.222-4.516)			2.494 ***	(1.504-3.878)			5.659	(2.976-10.761)

Adjusted for sex, age, educational attainment, working status, income level, tenure, and region; *$p < 0.05$, ***$p < 0.001$.

Table S2. Univariate analysis: Associations between housing cost burden, material hardship and depressive symptoms.

	Total (n = 1308)		Single-person (n = 761)		Non-single-person (n = 547)	
	OR	95% CI	OR	95% CI	OR	95% CI
Housing cost burden → material hardship	1.931 ***	(1.372-2.717)	2.040 **	(1.368, 3.043)	2.206*	(1.102, 4.415)
Housing cost burden → depressive symptoms	1.453	(0.966, 2.188)	1.730*	(1.070, 2.798)	1.224	(0.557, 2.691)

*$p < 0.05$, ***$p < 0.001$.
